# Evaluating the Statistical Fragility of Comparative Studies on Autografts for Pediatric ACL Reconstruction

**DOI:** 10.1177/23259671241313472

**Published:** 2025-02-06

**Authors:** Gurbinder Singh, Sergei O. Alexeev, Patrick Haugh, Ryan T. Halvorson, Dean Wang, Nirav K. Pandya, Brian T. Feeley

**Affiliations:** * Department of Orthopaedic Surgery, University of California–San Francisco, San Francisco, California, USA; † University of South Carolina School of Medicine, Columbia, South Carolina, USA; ‡ Department of Orthopaedic Surgery, University of California–Irvine, Orange, California, USA; § Department of Orthopaedic Surgery, University of California–San Francisco, San Francisco, California, USA; Investigation performed at the University of California–San Francisco, San Francisco, California, USA

**Keywords:** anterior cruciate ligament reconstruction, fragility index, fragility quotient, statistical fragility

## Abstract

**Background:**

The literature presents conflicting findings regarding outcomes after pediatric anterior cruciate ligament reconstruction (ACLR) with various autograft options, reflecting a lack of consensus on the standard of practice. Fragility analyses may assist in evaluating the statistical robustness of these studies.

**Purpose:**

To evaluate the statistical fragility of comparative studies in pediatric ACLR through the fragility index (FI) and fragility quotient (FQ), as well as qualitative factors such as outcome type, outcome significance, and patients lost to follow-up.

**Study Design:**

Systematic review; Level of evidence, 4.

**Methods:**

A systematic review conducted in accordance with the PRISMA (Preferred Reporting Items for Systematic Reviews and Meta-Analyses) guidelines identified 1139 studies in the PubMed and Embase databases that met the search criteria; ultimately, 6 studies were selected for inclusion. A total of 32 comparative outcomes were assessed for fragility across the 6 studies. Descriptive statistics were employed to summarize the fragility data and generate subgroup comparisons.

**Results:**

The mean FI was 1.5, and the mean reverse FI was 3.19 (*P* < .01); the mean FQ was 0.0064, and the mean reverse FQ was 0.028 (*P*≤ .0001). No significant difference was found in the FIs between objective outcomes and patient-reported outcomes (*P* = .418). These findings suggested that a comparable number of patients would need to transition from a nonevent to an event to alter a statistically significant result to a nonsignificant one. The FI was lower than the estimated number of patients lost to follow-up for 30 of the 32 outcomes (93.7%).

**Conclusion:**

Comparative studies on pediatric ACLR autograft outcomes displayed vulnerability when assessed using fragility metrics, indicating a lack of statistically robust data. The findings revealed that many reported outcomes are fragile and may require further investigation. Future research should incorporate fragility analyses—especially in studies with long-term follow-ups—to enhance the reliability of conclusions regarding optimal graft selection in pediatric ACLR.

Numerous clinical studies have been conducted to compare the outcomes of anterior cruciate ligament (ACL) reconstruction (ACLR) using various graft options in pediatric patients.^24^ ACLR in the pediatric population differs from that in the adult population since surgical techniques must be utilized to minimize the risk of growth disturbance while still providing tibiofemoral stability during pivoting sports activities.^28^ Comparative pediatric ACLR autograft studies have provided valuable evidence regarding the efficacy and safety of patellar tendon, quadriceps tendon, and hamstring tendon autografts in the pediatric population.^5,7,22,24,34^ However, it is essential to critically assess the stability of the conclusions drawn from these studies, as the significance of the study results may be influenced by a small number of outcome events. Critically assessing the stability of study conclusions is particularly important given the current controversy regarding pediatric ACLR graft selection and the equivocal nature of outcomes among different autografts.^15,21,37^ The equivocal outcomes of pediatric ACLR graft selection can be quantified using a statistical measure called the *fragility index* (FI).

Since its introduction^11^ in 1990, the concept of the FI has gained recognition as a valuable tool for evaluating the fragility of research studies within various medical disciplines. By quantifying the number of events or outcomes required to nullify the statistical significance of a result, the FI indicates the robustness of the study’s results.^11,24,30^ The *reverse fragility index* (RFI) offers insights into how many more events would need to be reversed in a study for it to acquire statistical significance (most commonly, *P* < .05), which may potentially be utilized for identifying the fragility of negative results. This analysis enables a more nuanced interpretation of hypothesis test results, with a low FI or RFI suggesting that a result may be underpowered or more likely to be attributable to chance.^11,35,36^

Although several studies have investigated the outcomes of ACLR using different autograft types in pediatric patients, the FI of these studies has not been explored.^8,22,33^ Exploring the statistical validity of this body of pediatric ACLR literature is critical, as multiple novel surgical techniques are rapidly being developed, all of which have implications with regard to potential growth disturbance and rerupture rate.

This study aimed to assess the vulnerability and reliability of current research on pediatric ACLR graft choices by evaluating the FI of comparative clinical trials. We hypothesized that the FIs would reveal significant fragility, underscoring the need for careful consideration of the robustness of these research conclusions.

## Methods

Primary research published between 2010 and 2023 that investigated comparative outcomes of different autograft types for ACLR in pediatric patients was queried for this study. The initial search strategy involved a well-established methodological querying of the PubMed and Embase online databases for studies related to the ACL or ACLR in pediatric patients.^9,12,13,29,38^ The titles and abstracts of the retrieved studies were screened by 3 authors (G.S., S.A., and P.H.) for relevance to pediatric ACLR utilizing autografts. Studies were excluded if they met any of the following criteria: (1) no dichotomous outcomes generated, or no *P* values or statistical significance reported; (2) not a pediatric study, or no report of outcomes in a pediatric population; (3) not an autograft study with differential autograft outcomes, (4) not primary research; (5) a cadaveric study; or (6) if the study used population databases, national registries, or cross-sectional data.

### Fragility Metrics

To assess the stability and reliability of the reported outcomes in these studies, the mean FI, *fragility quotient* (FQ), RFI, and *reverse fragility quotient* (RFQ) were calculated for each study, as well as each outcome measured. To determine the FI for each outcome, an established trial-and-error method was employed.^9,12,13,29^ Additionally, the FQ and RFQ were calculated for each outcome by dividing the FI or RFI by the number of patients included in the study. The FQ represents the proportion of events in the overall sample size that would have been reversed to generate a nonsignificant result.

### Outcomes Assessed

The outcomes were grouped into objective outcomes—including graft failure and postoperative complications such as arthrofibrosis—and clinical or patient-reported outcomes such as return to play. In addition, the reported *P* value for each outcome was verified for accuracy using the 2-tailed Fisher exact test. Outcomes with a listed significance discordant with the calculated Fisher test were assigned an FI or RFI of 0 because no results were needed to be flipped for post hoc calculated significance to change. An FI of 0 may be generated in the setting of an analysis using a different statistical test than the Fisher exact test for a dichotomous outcome.^1^ In this review we only analyzed dichotomous outcomes; thus, the patient-reported outcomes analyzed fell into dichotomous categories (eg, whether or not patients met functional recovery based on Knee injury and Osteoarthritis Outcome scores^15^).

The mean FI, FQ, RFI, and RFQ for all included outcome events were calculated along with their interquartile ranges. Three subgroups were analyzed for significant differences using independent *t* tests at a 95% CI: (1) graft failure or arthrofibrosis outcomes versus clinical or patient-reported outcomes; (2) significant (*P* < .05) versus nonsignificant (*P*≥ .05) outcomes; and (3) outcomes for which the FI or RFI was less than the estimated number of patients lost to follow-up (LTFU).

The data analysis was conducted utilizing Excel Version 16.80 (Microsoft) and R programming language Version 4.3.2 (R Core Team). Descriptive statistics were employed to summarize the fragility data and generate subgroup comparisons.

## Results

**Figure 1.**
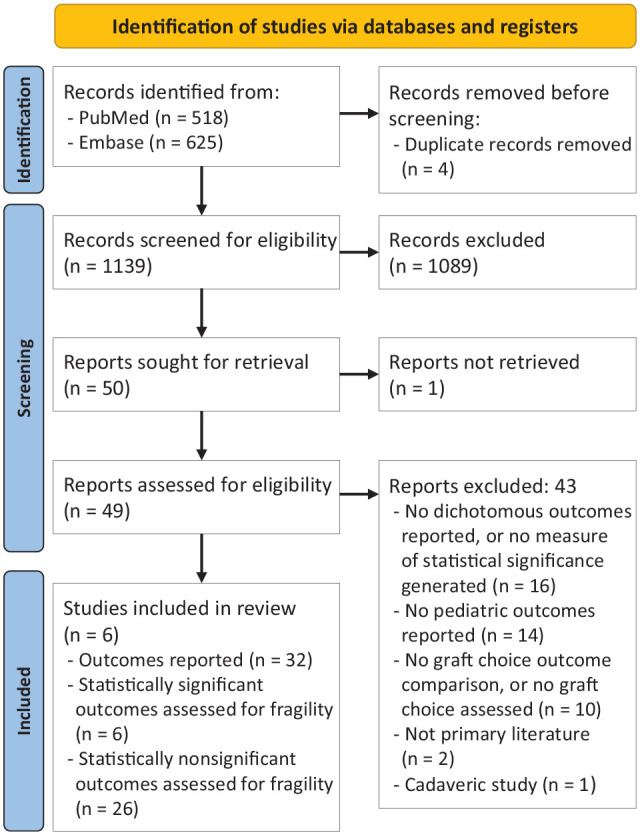
Identification of studies for inclusion via databases and registers.

A total of 1139 studies were initially screened, resulting in 50 studies meeting the initial search criteria. From this final pool, 6 studies were selected for the final analysis. The flow chart of study inclusion is depicted in Figure 1. In these studies, bone-patellar tendon-bone (BPTB), quadriceps tendon, hamstring tendon, and iliotibial band grafts were compared (Table 1).

**Table 1:** Characteristics of the Included Studies (N = 6)^*a*^

Lead Author (Year)	Study Type; LOE	Sample Size, n	Power Analysis Conducted	Graft Types Compared	Outcome Comparisons Analyzed, n	FI^*b*^	FQ^*b*^	RFI^*b*^	RFQ^*b*^
Britt^3^ (2020)	Retrospective case series; 4	71	Post hoc power analysis; underpowered (β = 0.8)	HT, BPTB	8	NA (0)	—	2.88 (8)	0.048
Ithurburn^15^ (2022)	Prospective cohort; 1	222	None described	HT, BPTB	2	1 (2)	0.011	NA (0)	NA
Kilkenny^18^ (2022)	Retrospective cohort; 3	358	None described	HT, BPTB	14	0 (1)	0	3.54 (13)	0.0222
Maheshwer^21^ (2023)	Retrospective case series; 4	482	None described	HT, QT, BPTB	3	3 (1)	0.0107	2.5 (2)	0.0104
Morgan^25^ (2016)	Retrospective case series; 4	242	None described	HT, BPTB	3	NA (0)	—	3 (3)	0.0124
Nwachukwu^26^ (2011)	Retrospective case series; 4	902	None described	HT, BPTB, ITB	2	2 (2)	0.0032	NA (0)	NA
Overall means	—	—	—	—	5.33	1.5	0.0064	3.192	0.0276

a BPTB, bone-patellar tendon-bone; FI, fragility index; FQ, fragility quotient; HT, hamstring tendon; ITB, iliotibial band; LOE, level of evidence; NA, not available; QT, quadriceps tendon; RFI, reverse fragility index; RFQ, reverse fragility quotient.

b Data are presented as means. The number of values used to calculate the mean FI and RFI are included in parentheses.

The mean FIs of the included studies ranged from 0 to 3, with an overall mean of 1.5, indicating that on average, <2 events would annul the statistical significance of the reported outcomes if changed to nonevents. The mean FQ ranged from 0 to 0.01, with an overall mean of 0.006, suggesting that, on average, around 0.6% of the sample size would need to be altered to nullify the statistical significance of the outcome (Table 1). The mean RFI and RFQ were calculated to measure the fragility of nonsignificant results with reported *P*≥ .05. Calculated mean RFIs ranged from 1 to 7, with an overall average of 3.19. The mean RFQ was 0.042 (Table 1).

No difference was observed between the magnitude of fragility between graft complication or clinical versus functional or patient-reported outcomes (*P* = .418) (Table 2). This result suggested that the statistical fragility of patient-reported outcomes may not be significantly different from more concrete outcomes such as the proportion of ACL graft failure. Significant outcomes were found to be less robust (more fragile) than nonsignificant outcomes, as reflected by the smaller FI values for significant results and the larger RFI values for nonsignificant results (Table 2). Our analysis examining the relationship between the FI and the number of patients LTFU did not reveal a statistically significant difference between the subgroups of FI or RFI ≤ LTFU and the FI or RFI > LTFU (Table 2).

**Table 2:** Overall Fragility Data and Analysis of Subgroups^*a*^

Analysis	No. of Comparative Outcomes	Magnitude of FI	Magnitude of FQ
All trials	32	2.875 (1.75-4)	0.02364 (0.00946-0.04225)
Outcome type			
Graft failure or arthrofibrosis findings	17	2.8 (1-5)	0.02769 (0.00826-0.04225)
Return-to-sport or functional recovery metrics	15	2.941 (2-3)	0.01961 (0.01163-0.02266)
*P* value		0.4177	0.09675
Reported outcome significance			
Significant outcomes (*P*≤ .05; FI values)	6	1.5 (1-2)	0.00639 (0.00287-0.00975)
Nonsignificant outcomes (*P* > .05; RFI values)	26	3.192 (2-4.75)	0.02762 (0.01163-0.04225)
*P*		.00656	<.001
Comparing outcome fragility with estimated patient LTFU			
FI or RFI ≤ patients LTFU	22	2.818 (1.25-4)	0.02675 (0.01240-0.04225)
FI or RFI > patients LTFU	3	5.333 (4.5-7)	0.03953 (0.01859-0.05516)
*P*		.1338	.3095

a Data are presented as mean (IQR). FI, fragility index; FQ, fragility quotient; IQR, interquartile range; LTFU, lost to follow-up.

## Discussion

The mean magnitude of fragility indices for all comparative outcomes was 2.875, indicating that a mean of <3 events would need to be reversed to alter the statistical significance of most findings within these studies of pediatric autograft ACLR. An approximate FI of 3 suggests the similar vulnerability of the conclusions in pediatric orthopaedic ACLR studies to the previous orthopaedic literature reporting similar FI values in sports medicine studies,^17^ and studies focusing on surgical techniques and rehabilitation in pediatric ACL tears.^9,23,33^ The American Academy of Orthopaedic Surgeons guidelines indicate that an FI ≥2 is desirable.^6^ Although the fragility of the negative findings in these studies met the desired standard, the positive findings, which achieved statistical significance (*P* < .05), did not. The mean FI for positive findings was 1.5, indicating that, on average, reversing the outcome of <2 patients would change the significance of the study. Furthermore, the highest FI observed was 3, meaning that in the most fragile positive results, reversing the outcomes of just 3 patients would eliminate statistical significance.

Notably, none of the included studies conducted an a priori power analysis, and only 1 study^3^ conducted a post hoc analysis. Britt et al^3^ describe conducting a power analysis that was underpowered at β = 0.8. Power analyses are a crucial component of strong comparative clinical studies that help determine minimal sample sizes^31^ and can help guide researchers to reduce fragility, ensure adequate sensitivity, estimate effect size, and assess the risk of type 2 errors in their final analysis.^2,4,32^ Therefore, we suggest orthopaedic researchers perform a priori power analyses during the study design phase and conduct post hoc analyses to ensure the validity of their findings. When considering the type of outcome, our study revealed no significant difference in fragility between groups of outcomes measuring concrete events such as graft rerupture and patient-reported outcomes such as return to play and functional recovery (Table 2). Patient-reported outcomes have previously faced criticism for their perceived lack of precision, unsubstantiated correlations with overall outcomes, increased susceptibility to recall bias, and inherent challenges with interpretation.^10,14,19,20,27^ However, through a fragility analysis, patient-reported outcomes can be compared with objective outcomes to help orthopaedic surgeons assess their congruence, evaluate the robustness and quality of patient-reported outcomes, and inform patient-centered clinical decision-making.

The accuracy of patient-reported outcomes is also supported after clinical rehabilitation of ACL tears within the nonoperative setting,^16^ suggesting that the inclusion of both concrete and patient-reported outcomes can provide an accurate assessment of treatment outcomes and contribute to the overall validity and clinical applicability of research findings. The characterization of FI and FQ by previous studies demonstrates the moderate vulnerability of the patient-reported outcomes in pediatric ACLR relative to other areas of orthopaedic research.^18,25^ The most statistically robust conclusions that demonstrate significance drawn from this body of literature are from Maheshwer et al,^21^ where an FI of 3 was generated from their analysis comparing the higher rate of retear in hamstring autograft ACLR to BPTB autograft at >2 years of follow-up. This finding suggests that only 3 event reversals would be needed to change the outcome’s statistical significance, indicating moderate fragility. An FI of 0 was generated in analyzing retear rates in 13- to 15-year-old patients who received either hamstring or BPTB autografts,^18^ signifying that even a single event change would affect the study’s conclusions, demonstrating extreme fragility. The context provided by these results is critical for our study, as it underscores the variability in statistical robustness across different studies. For patient management, these findings highlight the necessity for clinicians to critically evaluate the robustness of the evidence when making decisions about autograft selection for pediatric ACLR. The fragility of some studies suggests that clinical decisions should not rely solely on statistically significant findings but also consider the FI and other qualitative factors to ensure more reliable outcomes.

Among the nonsignificant results, notable findings emerged from studies such as Morgan et al^25^ and Kilkenny et al.^18^ Morgan et al reported comparable rerupture rates between BPTB and hamstring autografts, yielding an FI of 7. Similarly, Kilkenny et al observed no disparity in outcomes among 13- to 15-year-old patients who underwent BPTB or hamstring autograft repair, resulting in an FI of 7. Morgan et al reported the lowest FI in our analysis, scoring 0, when investigating the 15-year follow-up of BPTB versus hamstring graft repair and contralateral ACL rupture rates.

### Limitations

This study has several limitations. One such limitation is that the FI was not able to be calculated for nondichotomous data. Therefore, several studies and outcomes that examined nondichotomous outcome data in the setting of pediatric autograft ACLR were excluded, as these were unable to be examined with fragility methodology. The outcomes were grouped into graft rupture or arthrofibrosis findings, or clinical and patient-reported outcomes, which was a post hoc analysis performed after the conclusion of the literature search. This review provides a critical outlook on the strength of the studies examining autograft choice in pediatric ACLR, but as autograft choices exhibit individualized indications, the randomization of graft choice was not considered here. We primarily focused on evaluating population-level analysis, neglecting other patient-specific factors such as age, skeletal maturity, and activity level, which play a crucial role in determining tailored treatment approaches.^22,28,37^ Additionally, the lack of long-term follow-up studies limited our understanding of the durability and functional outcomes associated with different graft options.

## Conclusion

The findings of comparative studies investigating outcomes of pediatric ACLR with different autografts were found to be subject to vulnerability when evaluated using fragility metrics. There was a lack of statistically robust data adequately describing the similarities and differences in outcomes between various pediatric ACLR autograft choices. Many outcomes in the literature may be statistically fragile and may require further investigation. Future comparative study analyses should consider evaluating pediatric ACLR studies with long-term follow-ups with fragility metrics to ensure more reliable conclusions.

## References

[1] Andrade C. The use and limitations of the fragility index in the interpretation of clinical trial findings. J Clin Psychiatry. 2020;81(2):20f13334. doi:10.4088/JCP.20f13334 32237291

[2] Baer BR Gaudino M Fremes SE Charlson M Wells MT. The fragility index can be used for sample size calculations in clinical trials. J Clin Epidemiol. 2021;139:199-209. doi:10.1016/j.jclinepi.2021.08.010 34403756 PMC8665025

[3] Britt E Ouillette R Edmonds E , et al. The challenges of treating female soccer players with ACL injuries: hamstring versus bone-patellar tendon-bone autograft. Orthop J Sports Med. 2020;8(11):2325967120964884. doi:10.1177/2325967120964884 PMC7708716 33294473

[4] Caldwell JME Youssefzadeh K Limpisvasti O . A method for calculating the fragility index of continuous outcomes. J Clin Epidemiol. 2021;136:20-25. doi:10.1016/j.jclinepi.2021.02.023 33684509

[5] Carrozzo A Monaco E Saithna A , et al. Clinical outcomes of combined anterior cruciate ligament reconstruction and lateral extra-articular tenodesis procedures in skeletally immature patients: a systematic review from the SANTI Study Group. J Pediatr Orthop. 2023;43(1):24-30. doi:10.1097/BPO.0000000000002236 35980761

[6] Checketts JX Scott JT Meyer C Horn J Jones J Vassar M. The robustness of trials that guide evidence-based orthopaedic surgery. JBJS. 2018;100(12):e85. doi:10.2106/JBJS.17.01039 29916938

[7] Cordasco FA Hidalgo Perea S Uppstrom TJ , et al. Quadriceps tendon anterior cruciate ligament reconstruction in skeletally immature patients: 3-year clinical and patient-reported outcomes. Am J Sports Med. 2024;52(9):2230-2236. doi:10.1177/03635465241255641 38877730

[8] DeFazio MW Curry EJ Gustin MJ , et al. Return to sport after ACL reconstruction with a BTB versus hamstring tendon autograft: a systematic review and meta-analysis. Orthop J Sports Med. 2020;8(12):1-15. doi:10.1177/2325967120964919 PMC7745570 33403206

[9] Ehlers CB Curley AJ Fackler NP Minhas A Chang ES. The statistical fragility of hamstring versus patellar tendon autografts for anterior cruciate ligament reconstruction: a systematic review of comparative studies. Am J Sports Med. 2021;49(10):2827-2833. doi:10.1177/0363546520969973 33211555

[10] Ekanayake CD DeMik DE Glass NA Kotseos C Callaghan JJ Ratigan BL. Comparison of patient-reported outcomes and functional assessment using a marker-less image capture system in end-stage knee arthritis. J Arthroplasty. 2022;37(11):2158-2163. doi:10.1016/j.arth.2022.05.039 35644460

[11] Feinstein AR. The unit fragility index: an additional appraisal of “statistical significance” for a contrast of two proportions. J Clin Epidemiol. 1990;43(2):201-209. doi:10.1016/0895-4356(90)90186-S 2303850

[12] Forrester LA McCormick KL Bonsignore-Opp L , et al. Statistical fragility of surgical clinical trials in orthopaedic trauma. J Am Acad Orthop Surg Glob Res Rev. 2021;5(11):1-6. doi:10.5435/JAAOSGlobal-D-20-00197 34807889 PMC8608260

[13] Grolleau F Collins GS Smarandache A , et al. The fragility and reliability of conclusions of anesthesia and critical care randomized trials with statistically significant findings: a systematic review. Crit Care Med. 2019;47(3):456-462. doi:10.1097/CCM.0000000000003527 30394920

[14] Halawi MJ Jongbloed W Baron S Savoy L Cote MP Lieberman JR . Patient-reported outcome measures are not a valid proxy for patient satisfaction in total joint arthroplasty. J Arthroplasty. 2020;35(2):335-339. doi:10.1016/j.arth.2019.09.033 31611162

[15] Ithurburn MP Barenius B Thomas S Paterno MV Schmitt LC. Few young athletes meet newly derived age- and activity-relevant functional recovery targets after ACL reconstruction. Knee Surg Sports Traumatol Arthrosc. 2022;30(10):3268-3276. doi:10.1007/s00167-021-06769-4 34762143

[16] James EW Dawkins BJ Schachne JM , et al. Early operative versus delayed operative versus nonoperative treatment of pediatric and adolescent anterior cruciate ligament injuries: a systematic review and meta-analysis. Am J Sports Med. 2021;49(14):4008-4017. doi:10.1177/0363546521990817 33720764

[17] Khan M Evaniew N Gichuru M , et al. The fragility of statistically significant findings from randomized trials in sports surgery: a systematic survey. Am J Sports Med. 2017;45(9):2164-2170. doi:10.1177/0363546516674469 27895038

[18] Kilkenny CJ Hurley ET Hogan RE , et al. Return to play in paediatric & adolescent patients following anterior cruciate ligament reconstruction. Knee. 2022;37:87-94. doi:10.1016/j.knee.2022.05.013 35728392

[19] Lam KC Marshall AN Snyder Valier AR. Patient-reported outcome measures in sports medicine: a concise resource for clinicians and researchers. J Athl Train. 2020;55(4):390-408. doi:10.4085/1062-6050-171-19 32031883 PMC7164564

[20] Lee MK Zaniletti I Larson DR Lewallen DG Berry DJ Maradit Kremers H. Nuts and bolts of patient-reported outcomes in orthopaedics. J Arthroplasty. 2023;38(4):616-621. doi:10.1016/j.arth.2022.11.011 36481287 PMC10010940

[21] Maheshwer B Paliobeis A Halkiadakis P Konda S Calcei JG Voos JE. Anterior cruciate ligament tears in the adolescent population: injury demographics and risk of reinjury among high school athletes. J Pediatr Orthop. 2023;43(10):591-597. doi:10.1097/BPO.0000000000002505 37728131

[22] Marx RG Hsu J Fink C Eriksson K Vincent A van der Merwe WM. Graft choices for paediatric anterior cruciate ligament reconstruction: State of the art. J ISAKOS. 2023;8(3):145-152. doi:10.1016/j.jisako.2023.01.001 36646171

[23] Matsuzaki Y Chipman DE Hidalgo Perea S Green DW. Unique considerations for the pediatric athlete during rehabilitation and return to sport after anterior cruciate ligament reconstruction. Arthrosc Sports Med Rehabil. 2022;4(1):e221-e230. doi:10.1016/j.asmr.2021.09.037 PMC8811511 35141555

[24] Monaco E Carrozzo A Saithna A , et al. Isolated ACL reconstruction versus ACL reconstruction combined with lateral extra-articular tenodesis: a comparative study of clinical outcomes in adolescent patients. Am J Sports Med. 2022;50(12):3244-3255. doi:10.1177/03635465221118377 36113005

[25] Morgan MD Salmon LJ Waller A Roe JP Pinczewski LA. Fifteen-year survival of endoscopic anterior cruciate ligament reconstruction in patients aged 18 years and younger. Am J Sports Med. 2016;44(2):384-392. doi:10.1177/0363546515623032 26759030

[26] Nwachukwu BU McFeely ED Nasreddine A , et al. Arthrofibrosis after anterior cruciate ligament reconstruction in children and adolescents. J Pediatr Orthop. 2011;31(8):811-817. doi:10.1097/BPO.0b013e31822e0291 22101657

[27] Ott NA Barber CL Jordan SE. Trends in Patient-Reported Outcomes Measurement Information System (PROMIS) utilization in orthopedic sports medicine: a scoping review. Ochsner J. 2023;23(4):304-314. doi:10.31486/toj.23.0054 38143549 PMC10741822

[28] Pandya NK Feeley B Wong S. Over-the-top versus all-epiphyseal technique for physeal sparing ACL reconstruction. Orthop J Sports Med. 2019;7(suppl 3):2325967119S0014. doi:10.1177/2325967119s00147 PMC6440065 30944841

[29] Parisien RL Dashe J Cronin PK Bhandari M Tornetta P. Statistical significance in trauma research: too unstable to trust? J Orthop Trauma. 2019;33(12):E466-E470. doi:10.1097/BOT.0000000000001595 31356443

[30] Peterson DC Ayeni OR. Pediatric anterior cruciate ligament reconstruction outcomes. Curr Rev Musculoskelet Med. 2016;9(4):339-347. doi:10.1007/s12178-016-9358-3 27709485 PMC5127938

[31] Raittio L Reito A. Assessing variability and uncertainty in orthopedic randomized controlled trials. Acta Orthop. 91(4):479-484. doi:10.1080/17453674.2020.1755932 PMC8023899 32316873

[32] Reito A Raittio L Helminen O. Fragility Index, power, strength and robustness of findings in sports medicine and arthroscopic surgery: a secondary analysis of data from a study on use of the Fragility Index in sports surgery. PeerJ. 2019;7:e6813. doi:10.7717/peerj.6813 PMC6536113 31179168

[33] Tang C Kwaees TA Accadbled F Turati M Green DW Nicolaou N. Surgical techniques in the management of pediatric anterior cruciate ligament tears: Current concepts. J Child Orthop. 2023;17(1):12-21. doi:10.1177/18632521221149059 36755552 PMC9900020

[34] Turati M Caliandro M Gaddi D , et al. Clinical outcomes and complications after anterior cruciate ligament reconstruction with bone–patellar tendon–bone in patient Tanner 3 and 4: a systematic review. Eur J Orthop Surg Traumatol. 2023;33(6):2191-2199. doi:10.1007/s00590-022-03402-z 36307618 PMC10368545

[35] Walsh M Srinathan SK McAuley DF , et al. The statistical significance of randomized controlled trial results is frequently fragile: a case for a Fragility Index. J Clin Epidemiol. 2014;67(6):622-628. doi:10.1016/j.jclinepi.2013.10.019 24508144

[36] Wu HH Liu M Patel KR , et al. Impact of academic collaboration and quality of clinical orthopaedic research conducted in low- and middle-income countries. SICOT-J. 2017;3. doi:10.1051/sicotj/2016042 PMC5278648 28134090

[37] Zakharia A Lameire DL Abdel Khalik H , et al. Quadriceps tendon autograft for pediatric anterior cruciate ligament reconstruction results in promising postoperative function and rates of return to sports: a systematic review. Knee Surg Sports Traumatol Arthrosc. 2022;30(11):3659-3672. doi:10.1007/s00167-022-06930-7 35445330

[38] Zhang H Li J Zeng W. Frequent fragility of randomized controlled trials for HCC treatment. BMC Cancer. 2021;21(1):1-7. doi:10.1186/s12885-021-08133-8 33836710 PMC8034173

